# Contractile Activity of Myotubes Derived from Human Induced Pluripotent Stem Cells: A Model of Duchenne Muscular Dystrophy

**DOI:** 10.3390/cells10102556

**Published:** 2021-09-27

**Authors:** Kantaro Yoshioka, Akira Ito, Masanobu Horie, Kazushi Ikeda, Sho Kataoka, Keiichiro Sato, Taichi Yoshigai, Hidetoshi Sakurai, Akitsu Hotta, Yoshinori Kawabe, Masamichi Kamihira

**Affiliations:** 1 Department of Chemical Engineering, Faculty of Engineering, Kyushu University, Fukuoka 819-0395, Japan; kyoshioka@kyudai.jp (K.Y.); 1te17459r@kyudai.jp (S.K.); 310k16081230@gmail.com (K.S.); taichi800@gmail.com (T.Y.); kawabe@chem-eng.kyushu-u.ac.jp (Y.K.); 2 Department of Chemical Systems Engineering, School of Engineering, Nagoya University, Nagoya 464-8603, Japan; ito.akira@material.nagoya-u.ac.jp; 3 Division of Biochemical Engineering, Radioisotope Research Center, Kyoto University, Kyoto 606-8501, Japan; horie.masanobu.4z@kyoto-u.ac.jp; 4 Graduate School of Systems Life Sciences, Kyushu University, Fukuoka 819-0395, Japan; kazushi.ikeda@kyudai.jp; 5 Center for iPS Cell Research and Application, Kyoto University, Kyoto 606-8507, Japan; hsakurai@cira.kyoto-u.ac.jp (H.S.); akitsu.hotta@cira.kyoto-u.ac.jp (A.H.)

**Keywords:** Duchenne muscular dystrophy, human induced pluripotent stem cell, myotube, contractile activity, CRISPR/Cas9

## Abstract

Duchenne muscular dystrophy (DMD) is a genetic disorder that results from deficiency of the dystrophin protein. In recent years, DMD pathological models have been created using induced pluripotent stem (iPS) cells derived from DMD patients. In addition, gene therapy using CRISPR-Cas9 technology to repair the dystrophin gene has been proposed as a new treatment method for DMD. However, it is not known whether the contractile function of myotubes derived from gene-repaired iPS cells can be restored. We therefore investigated the maturation of myotubes in electrical pulse stimulation culture and examined the effect of gene repair by observing the contractile behaviour of myotubes. The contraction activity of myotubes derived from dystrophin-gene repaired iPS cells was improved by electrical pulse stimulation culture. The iPS cell method used in this study for evaluating muscle contractile activity is a useful technique for analysing the mechanism of hereditary muscular disease pathogenesis and for evaluating the efficacy of new drugs and gene therapy.

## 1. Introduction

Duchenne muscular dystrophy (DMD) is a genetic disorder characterized by progressive muscle weakness [1]. DMD patients die in their 30 s mainly due to cardiac and respiratory complications caused by muscle atrophy [2,3]. There is no effective treatment for DMD. Dystrophin, a huge protein composed of more than 3600 amino acids transcribed from 79 exons, is involved in DMD, and production of the protein is impaired by various mutations in the dystrophin gene [4,5]. The absence of dystrophin induces muscle cell death by apoptosis/necrosis and causes muscle atrophy [6]. The *mdx* mouse is commonly used as a DMD model to develop DMD treatments [6]. *Mdx* mice possess a nonsense mutation in exon 23 of the dystrophin gene, causing dystrophin deficiency [7]. However, unlike DMD, the mouse model does not completely mimic the pathology and physiology of DMD because there is little or no decline in muscle strength or lifespan. Recently, a model of DMD pathology was prepared by differentiating induced pluripotent stem (iPS) cells derived from DMD patients into myoblasts [8,9]. The iPS cells were differentiated into myoblasts by expressing the *MYOD1* gene [10,11], which is a master gene of muscle differentiation. MYOD1 is a transcription factor that regulates the differentiation of muscle progenitor cells into myoblasts and myotubes by controlling the expression of transcription factors, such as myogenin, involved in muscle differentiation [12]. Shoji et al. reported a method for inducing muscle differentiation of iPS cells by introducing a tetracycline-inducible *MYOD1* expression system, in which *MYOD1* gene expression can be controlled using doxycycline [9,11,13]. The myogenic differentiation efficiency of this method was high at 70–90%. In addition, Li et al. introduced this *MYOD1* expression system into iPS cells derived from a DMD patient lacking exon 44 of the dystrophin gene to induce muscle differentiation [8]. This confirmed that after muscle differentiation the cells did not express dystrophin protein, mimicking the pathology.

In recent years, gene therapy incorporating advances in genome editing technology using CRISPR-Cas9 has attracted much attention as a new approach for DMD treatment. CRISPR-Cas9 technology uses an endonuclease, Cas9, and a guide RNA complementary to a target gene sequence [14]. The complex of Cas9 and gRNA is recruited to the target gene sequence, and the target gene is cleaved. Then, a gene of interest on a donor vector can be knocked into the target site via the homologous recombination repair pathway. Using CRISPR-Cas9 technology, Li et al. repaired the dystrophin gene lacking exon 44 in iPS cells derived from DMD patients [8]. Then, by introducing a tetracycline-inducible *MYOD1* expression system into the iPS cells, muscle differentiation was induced, and recovery of dystrophin protein expression was confirmed in myotubes. However, functionality of the myotubes after gene therapy, such as contractile activity of cells, has not been analysed in detail.

We have been developing drug evaluation systems using muscle contraction as an index [15,16]. Candidate drugs are added to cultured myotubes derived from a mouse myoblast cell line, C2C12, and myotube morphology, differentiation efficiency and contractile activity with or without a drug are evaluated in response to electrical pulse stimulation. Myotube contractile activity is not always correlated with cell morphology and differentiation efficiency [15]. In this study, we evaluated whether DMD gene therapy is effective in terms of muscle contraction. For this purpose, three human iPS cell lines, normal iPS cells derived from a healthy donor (N-iPS), DMD patient-derived iPS cells (D-iPS), and D-iPS repaired cells using CRISPR-Cas9 technology (R-iPS), were differentiated into myotubes by introducing a tetracycline-inducible *MYOD1* expression system. It is necessary to induce maturation of myotubes for the evaluation of muscle contractile activity; therefore, when the three cell lines were induced to differentiate into myotubes by adding retinoic acid, electrical stimulation culture [17] was performed to promote muscle maturation and myotube contractile activity was evaluated.

## 2. Materials and Methods

### 2.1. Cells and Cell Culture 

N-iPS cells were established from a human iPS cell line (clone ID: 409B2 [18]) by transfection with a plasmid encoding a tetracycline-inducible *MYOD1* expression unit [11]. D-iPS cells were established from iPS cells (clone ID: CiRA00111) derived from a DMD patient lacking exon 44 of the dystrophin gene by transfection with a plasmid encoding a tetracycline-inducible *MYOD1* expression unit [8]. R-iPS cells were established from DMD patient-derived iPS cells (clone ID: CiRA00111-CKI-C2), in which exon 44 of the dystrophin gene was knocked-in using the CRISPR/Cas9 system (clone ID: CiRA00111), by transfection with a plasmid encoding a tetracycline-inducible *MYOD1* expression unit [8]. Human iPS cells were cultured in StemFit AK02N medium (Ajinomoto Healthy Supply, Tokyo, Japan) supplemented with 10 µM Y-27632 (MedChem Express, Monmouth Junction, NJ, USA) and 0.5 µg/mL puromycin (Thermo Fisher Scientific, Waltham, MA, USA) for N-iPS cells [11] or 0.1 mg/mL neomycin (Sigma-Aldrich, St. Louis, MO, USA) for D-iPS and R-iPS cells [8] using 6-well plates (Thermo Fisher Scientific) coated with laminin (iMatrix-511; Nippi, Tokyo, Japan) at the seeding density of 6.5 × 10^4^ cells/well. Culture medium was changed with fresh medium without Y-27632 every day. Cells were cultured at 37°C in a 5% (*v*/*v*) CO_2_ incubator. Cells were harvested using Accutase (Innovative Cell Technologies, San Diego, CA, USA).

All experiments using human iPS cells were carried out in accordance with our institutional guidelines on Medical and Health Research on Human Subjects and were approved by the Ethics Committee for Clinical Research at the Kyushu University Hospital Campus (approval number 27–169).

### 2.2. Myotube Differentiation

Myotube differentiation of human iPS cells was performed as previously described [8,11]. On day 0, N-iPS cells, D-iPS and R-iPS cells were seeded at densities of 3.0 × 10^5^, 5.0 × 10^5^, and 1.0 × 10^5^ cells/well, respectively, onto 6-well plates coated with Matrigel (354230; Corning, New York, NY, USA). Since the growth rate differed depending on the type of iPS cells, the seeding cell density was changed so that cells reach semi-confluency during the same period. Cells were cultured in StemFit AK02N medium supplemented with 10 µM Y-27632, 0.1, 1.0 or 10 µM retinoic acid (RA) (Fujifilm Wako Pure Chemical, Osaka, Japan), and 0.5 µg/mL puromycin or 0.1 mg/mL neomycin. The next day, cells were cultured in ReproStem medium (ReproCELL, Yokohama, Japan) supplemented with the corresponding concentrations of RA and antibiotics. From day 2, doxycycline (Dox) (Sigma-Aldrich) was added to the medium at a concentration of 1.0 µg/mL. On day 3, the culture medium was changed to α-MEM (Invitrogen, Carlsbad, CA, USA) containing 5% knock-out serum replacement (Invitrogen), supplemented with Dox and RA. Cells were cultured for 14 days and the medium was changed with fresh medium every day.

### 2.3. Electrical Pulse Stimulation Culture during Myotube Differentiation

To apply electrical pulse stimulation (EPS) to myotubes derived from human iPS cells, cells were differentiated into myotubes as described in Section 2.2. EPS culture conditions were determined based on our previous study [17]. Cells were stimulated with electrical pulses from day 7 to day 14 with the conditions of 0.17 or 0.3 V/mm pulse-strength, 4 ms pulse-width and 1 Hz frequency. Electrical pulses generated by a function generator (NF Corporation, Kanagawa, Japan) were applied to the cells in dishes equipped with electrodes (C-Dish; IonOptix, Milton, MA, USA).

### 2.4. Immunostaining

Immunostaining of cells was performed according to our previous study with slight modifications [19]. Briefly, cell samples washed three times with phosphate buffered saline (PBS) were fixed with 4% paraformaldehyde (Nara Pathology Laboratory, Nara, Japan). After blocking with 1% bovine serum albumin (Fujifilm Wako Pure Chemical) in PBS, cells were incubated with mouse anti-α-actinin primary antibodies (EA-53; Sigma-Aldrich), followed by incubation with goat anti-mouse IgG secondary antibodies conjugated with Alexa Fluor 488 or 546 (Thermo Fisher Scientific). Cell nuclei were stained with 4’,6-diamidino-2-phenylindole (DAPI) (Sigma-Aldrich). The stained cells were observed under a BZ-9000 fluorescence microscope (Keyence, Osaka, Japan).

### 2.5. Morphological Analysis

α-Actinin-positive areas were measured in five fields of view in each of three wells using BZ-Analyzer software (Keyence). Widths of 10 myotubes were measured in each of three wells using BZ-Analyzer software.

### 2.6. Measurement of Myotube Contractile Activity

To investigate contractile activity of myotubes, human iPS cells were differentiated into myotubes as described in Section 2.2 and Section 2.3. On day 14, myotubes were stimulated with electrical pulses: 0.3 V/mm 4 ms and 1 Hz [17], and myotube movement measured. Electrical pulses were applied to the myotubes using C-Dishes. Myotube movement was recorded at a rate of 15 frames/s for 25 s at three positions on the bottom surface in each of three separate dishes using a BZ-9000 fluorescence microscope. To estimate the range of displacement, three myotubes displaying the highest contractile activity in each of three fields in three separate dishes were measured using motion analyser software (Keyence). Contracting myotube efficiency was measured by counting the number of contracting myotubes from 16 randomly selected myotubes in each of three fields in three separate dishes using a BZ-9000 fluorescence microscope.

### 2.7. Apoptosis Analysis

Cells were stained by the terminal transferase dUTP nick end labelling (TUNEL) method. Cell samples were stained as described in Section 2.4. The samples were then placed in Permeabilisation Buffer (Takara Bio, Kusatsu, Japan) on ice in the dark for 5 min and washed three times with PBS. Next, samples were allowed to react with TdT enzyme (Takara Bio) in Labeling Safe Buffer (Takara Bio) at 37 °C in the dark for 1 h. The ratio of TUNEL-positive cells to total number of nuclei in the α-actinin positive area was measured in three fields of view in each of three plates using BZ-Analyzer software (Keyence).

### 2.8. Addition of Ca^2+^ Chelator

D-iPS cells were differentiated into myotubes as described in Section 2.2 by EPS culture from day 7 with the following conditions: 0.3 V/mm, 4 ms, and 1 Hz. Ethylene glycol tetra-acetic acid (EGTA) (Dojindo Laboratories, Kumamoto, Japan) was added on day 9 for 24 h and the apoptosis of myotubes was analysed as described in Section 2.7.

### 2.9. Addition of Bax Inhibitor

D-iPS cells were differentiated into myotubes as described in Section 2.2 by EPS culture from day 7 with the following conditions; 0.3 V/mm, 4 ms, and 1 Hz. From day 9 to day 14, 0.1, 1.0, or 10 µM Bax inhibitor peptide V5 (MedChem Express), which is a cell-permeable synthetic peptide inhibitor of Bax conformational change and mitochondrial translocation, was added. Myotubes were analysed as described in Section 2.6 and Section 2.7.

### 2.10. Statistical Analysis

The Mann-Whitney rank sum tests were performed to obtain *p*-values. Values of *p* < 0.05 were considered statistically significant.

## 3. Results

### 3.1. Retinoic Acid Induces the Differentiation of Human iPS Cells into Myotubes

**Figure 1:**
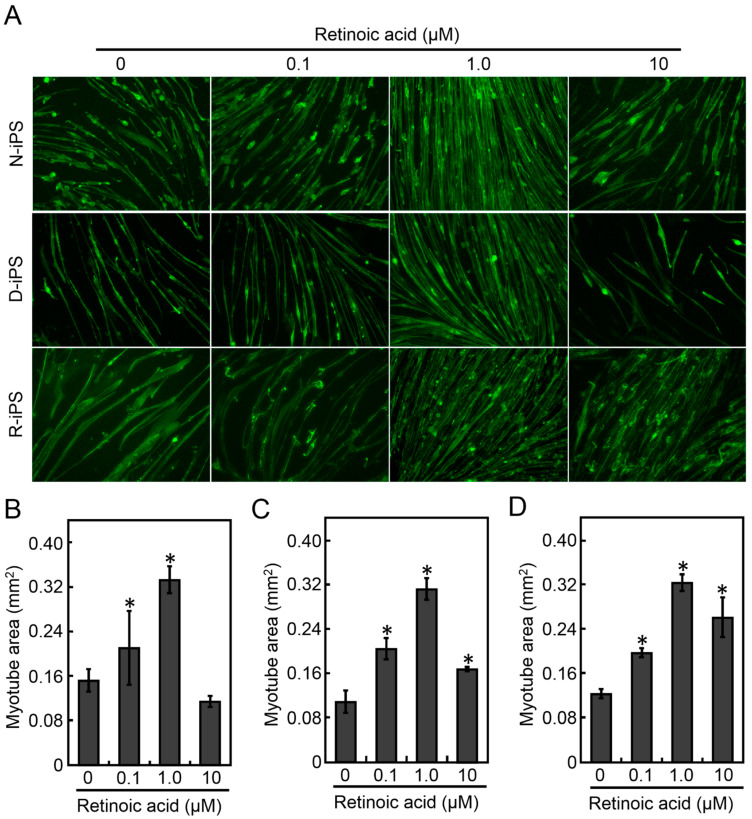
Morphological analysis of human iPS cell-derived myotubes cultured with varying concentrations of RA. (**A**) Fluorescence microscopy of myotubes stained with anti-α-actinin antibody. Scale bars, 100 μm. (B–D) Quantitative analysis of myotube area of N-iPS (**B**), D-iPS (**C**), and R-iPS (**D**) cell-derived myotubes. Data are expressed as the mean ± SD (*n* = 3). * *p* < 0.05 vs without RA.

To evaluate myotube function, it is necessary to efficiently differentiate iPS cells into myotubes. Retinoic acid (RA) promotes myogenic differentiation of P19 and human iPS cells [20]; therefore, RA was added to the culture medium. To optimise RA concentration, N-iPS, D-iPS and R-iPS cells were differentiated into myotubes in the presence of 0.1, 1.0, or 10 μM RA under the MYOD1 induction conditions, and the myotube morphology was analysed by immunostaining after 7 days of differentiation culture (Figure 1A). Immunostaining of α-actinin, a myogenic marker, revealed that RA increased the α-actinin- positive area for all cell lines (Figure 1B–D). In addition, the α-actinin-positive area was largest with 1.0 µM RA, which was approximately 0.32 mm^2^ for all cell lines (Figure 1B–D). There was no significant change in myotube width in any cell line with or without RA (Supplementary Figure S1). Furthermore, the promotion of myogenic maturation of iPS cell-derived myotubes by RA was confirmed by the expression of myogenic markers, although maturation level of D-iPS cell-derived myotubes was inferior (Supplementary Figure S2). These results indicate that RA increased the efficiency of myotube differentiation and that a RA concentration of 1.0 µM was optimal. Therefore, 1.0 µM RA was used to prepare myotubes for further experiments.

### 3.2. Electrical Pulse Stimulation Culture Improves the Contractile Function of Myotubes

**Figure 2:**
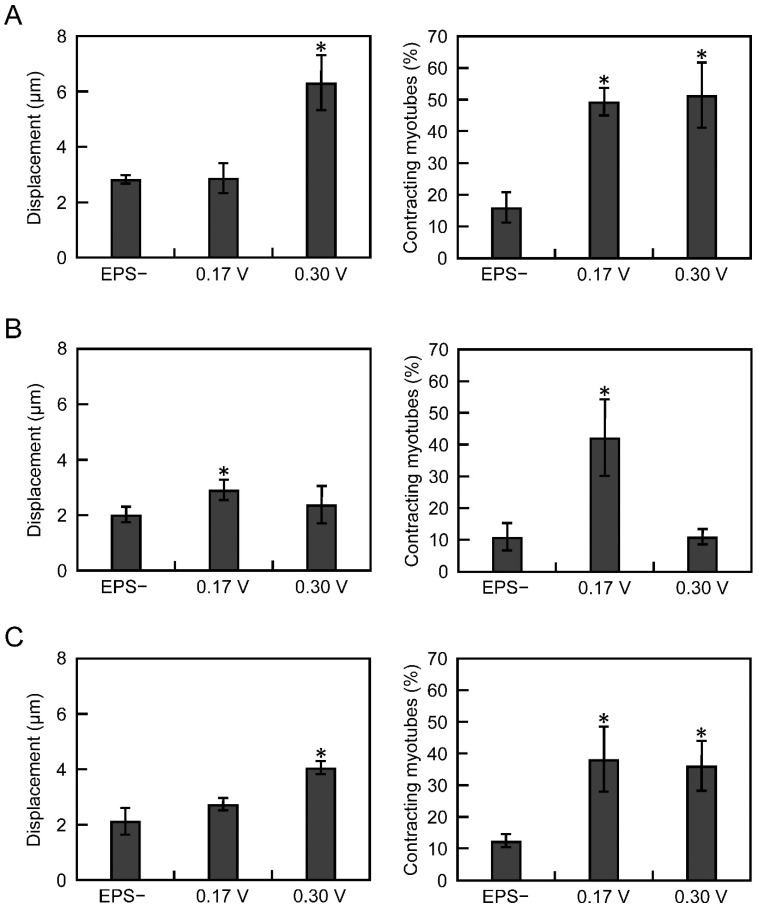
Contractile activity of human iPS cell-derived myotubes. Myotubes were cultured with or without electrical pulse stimulation (EPS). When EPS was applied to myotubes, two different voltages (0.17 and 0.30 V/mm) were used. Displacement (left) and the percentage of contracting myotubes (right) for N-iPS (**A**), D-iPS (**B**), and R-iPS (**C**) cell-derived myotubes were measured. Data are expressed as the mean ± SD (*n* = 3). * *p* < 0.05 vs without EPS (EPS−).

We previously reported that electric pulse stimulation (EPS) improved the contractility of three-dimensional muscle tissues derived from C2C12 cells [17]. Thus, to improve the contractile function of myotubes derived from iPS cells, EPS was applied during differentiation culture of iPS cells. Referring to our previous paper [17], cells differentiated from iPS cells were subjected to EPS culture from day 7 to day 14 under two voltage conditions, 0.17 or 0.3 V/mm (pulse width, 4 ms; pulse frequency, 1 Hz). Without EPS culture, myotube displacement was approximately 2 µm and contraction efficiency was approximately 10% for all the cell lines (Figure 2A–C). In contrast, in N-iPS and R-iPS cells, myotube displacement increased in the strong (0.3 V/mm) EPS culture, and contraction efficiency increased in the weak (0.17 V/mm) and strong (0.3 V/mm) EPS cultures (Figure 2A,C). However, in D-iPS cells, myotube displacement and contraction efficiency significantly increased with low-voltage EPS culture, and these increases were not observed with strong EPS culture (Figure 2B). These results indicate that iPS cell-derived myotubes were mature in EPS cultures and that gene repair using CRISPR-Cas9 technology can support the recovery of myotube contractile function.

### 3.3. iPS-Derived Myotube Apoptosis in Electrical Pulse Stimulation Cultures

**Figure 3:**
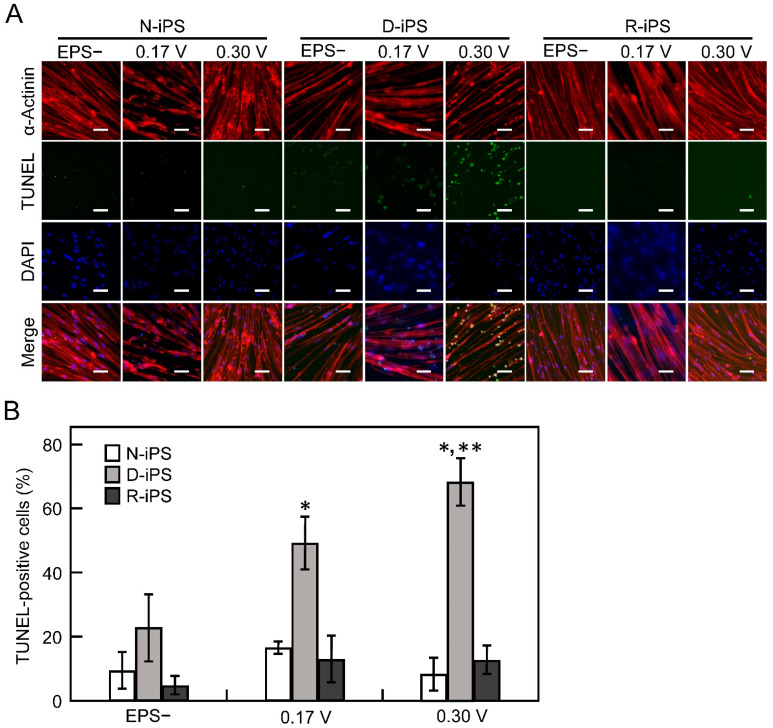
Apoptosis of human iPS cell-derived myotubes. Myotubes were cultured with or without electrical pulse stimulation (EPS). When EPS was applied to myotubes, two different voltages (0.17 and 0.30 V/mm) were used. (**A**) Fluorescence microscopy of myotubes stained with anti-α-actinin antibody (red) and DAPI (blue). Apoptotic cells were stained by the TUNEL assay (green). Scale bars, 50 μm. (**B**) Quantitative analysis of the percentage of TUNEL-positive myotubes derived from N-iPS, D-iPS, and R-iPS cells. Data are expressed as the mean ± SD (*n* = 3). * *p* < 0.05 vs without EPS (EPS−), ** *p* < 0.05 vs with EPS (0.17 V).

As shown in Figure 2B, the contractile activity of myotubes derived from D-iPS cells was unchanged with strong (0.3 V/mm) EPS culture. We hypothesised that apoptosis of D-iPS cell-derived myotubes with contractile function was induced in EPS cultures. Therefore, apoptosis of myotubes derived from each iPS cell line was analysed by the TUNEL method with or without EPS culture (Figure 3A). Less than 20% of cells were TUNEL-positive in N-iPS and R-iPS cells with and without EPS culture. For D-iPS cells, 23% of cells were TUNEL-positive without EPS culture (Figure 3B), but the proportion of TUNEL-positive cells increased to 49% and 68% in weak (0.17 V/mm) and strong (0.3 V/mm) EPS cultures, respectively (Figure 3B). These results indicate that apoptosis is induced by contraction in D-iPS cell-derived myotubes, and that contraction-induced apoptosis is prevented in myotubes derived from gene-repaired cells (R-iPS cells).

### 3.4. Apoptosis Is Suppressed by Chelating Ca^2+^

**Figure 4:**
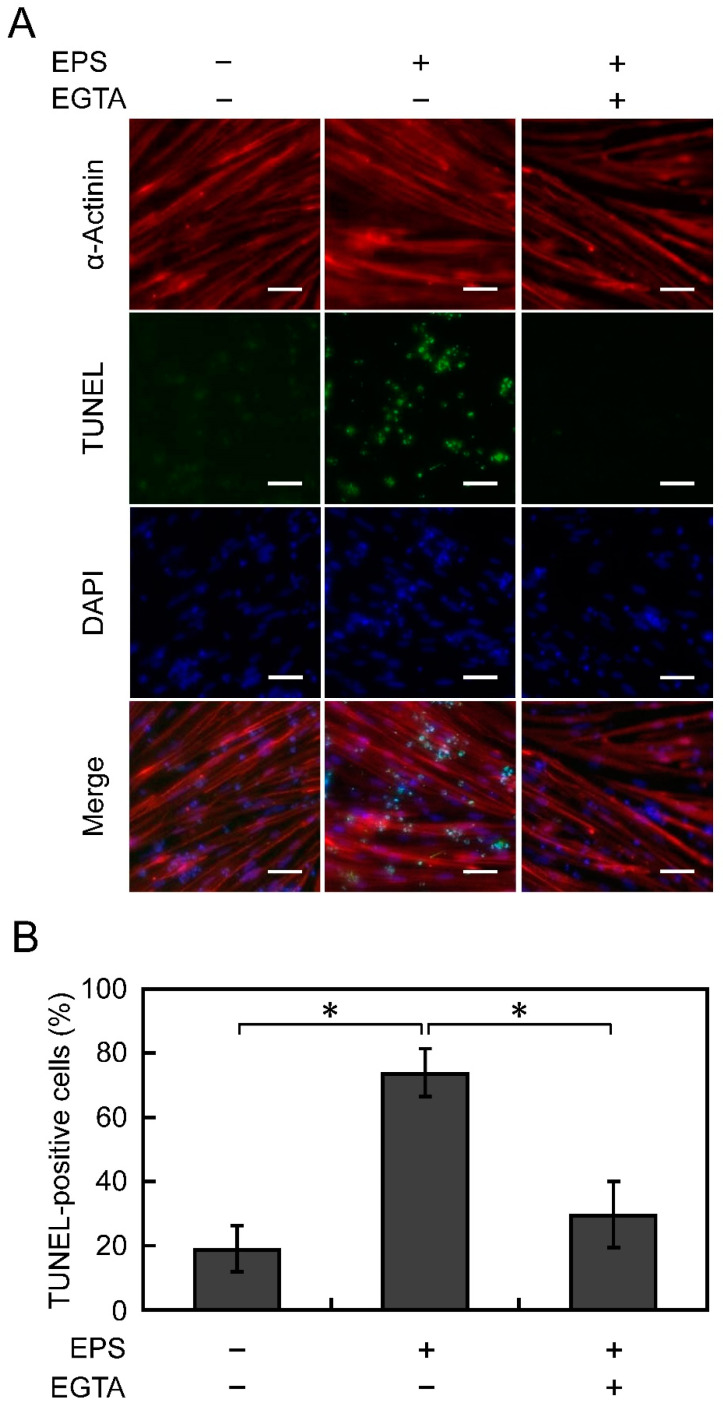
Effect of EGTA on apoptosis of D-iPS cell-derived myotubes. D-iPS cell-derived myotubes were cultured with or without electrical pulse stimulation (EPS) (0.30 V/mm) in the presence or absence of 1.0 mM EGTA. (**A**) Fluorescence microscopy of myotubes stained with anti-α-actinin antibody (red) and DAPI (blue). Apoptotic cells were stained by the TUNEL assay (green). Scale bars, 75 μm. (**B**) Quantitative analysis of the percentage of TUNEL-positive myotubes. Data are expressed as the mean ± SD (*n* = 3). * *p* < 0.05 vs without both EPS and EGTA (EPS–/EGTA–), or with both EPS and EGTA (EPS+/EGTA+).

We hypothesised that the apoptosis of D-iPS cell-derived myotubes under EPS culture was caused by excessive influx of Ca^2+^. Thus, EGTA, a Ca^2+^ chelator, was added to the culture medium to prevent excessive Ca^2+^ influx into cells, so as to inhibit myotube apoptosis. To optimise the duration of EGTA treatment, D-iPS cell-derived myotube apoptosis was analysed in EPS cultures (voltage, 0.3 V/mm; width, 4 ms; frequency, 1 Hz) on days 8, 10, 12, and 14. Few TUNEL-positive cells were observed on day 8 and the number of TUNEL-positive cells increased from day 10 (data not shown). Therefore, 1 mM EGTA was added to the medium for 24 h on day 9 and myotube apoptosis was analysed on day 10 (Figure 4A). The percentage of TUNEL-positive cells was 19% in normal culture without applying EPS, and it increased to 74% in EPS cultures. The percentage of TUNEL-positive cells in EPS cultures decreased to 30% with the addition of EGTA (Figure 4B). This result indicates that the D-iPS cell-derived myotube apoptosis was caused by excessive influx of Ca^2+^ upon contraction under the EPS culture conditions.

### 3.5. Apoptosis Is Suppressed by Inhibiting the Bax Pathway

**Figure 5:**
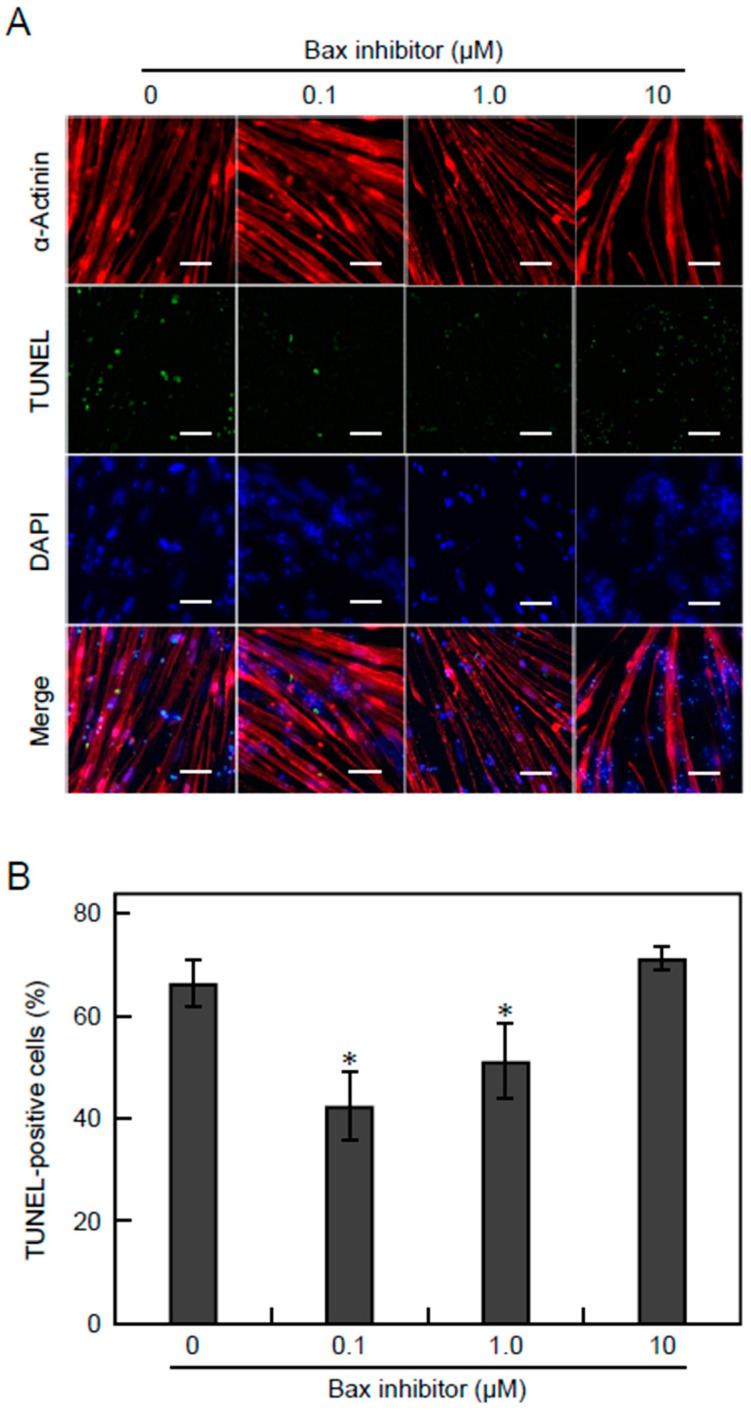
Effect of Bax inhibitor on apoptosis of D-iPS cell-derived myotubes. D-iPS cell-derived myotubes were cultured with EPS (0.30 V/mm). Bax inhibitor peptide was added to culture medium at varying concentrations (0.1, 1.0, and 10 μM). (**A**) Fluorescence microscopy of cells stained with anti-α-actinin antibody (red) and DAPI (blue). Apoptotic cells were stained by the TUNEL assay (green). Scale bar is 50 μm. (**B**) Quantitative analysis of the percentage of TUNEL-positive myotubes. Data are expressed as the mean ± SD (*n* = 3). * *p* < 0.05 vs without Bax inhibitor.

**Figure 6:**
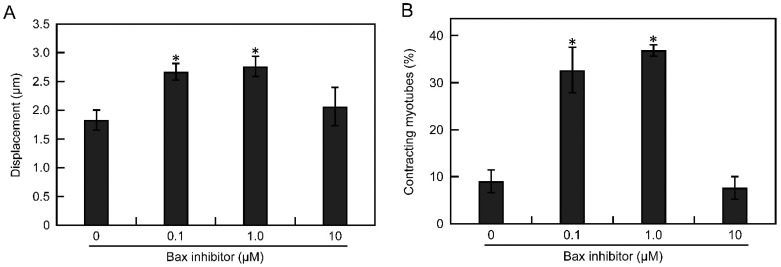
Contractile activity of D-iPS cell-derived myotubes cultured with EPS (0.30 V/mm) in the presence of Bax inhibitor. Bax inhibitor peptide was added to culture medium at varying concentrations (0.1, 1.0, and 10 μM). Displacement (**A**) and the percentage of contracting myotubes (**B**) were measured. Data are expressed as the mean ± SD (*n* = 3). * *p* < 0.05 vs without Bax inhibitor.

To suppress Ca^2+^-dependent apoptosis in EPS cultures, we added an inhibitor peptide of Bax, a regulator of Ca^2+^ homeostasis in the endoplasmic reticulum and mitochondria. On day 9 of differentiation, 0.1, 1.0, or 10 µM Bax inhibitor was added to the EPS culture medium (voltage, 0.3 V/mm; width, 4 ms; frequency, 1 Hz) of D-iPS cell-derived myotubes. On day 14, myotube apoptosis was analysed by TUNEL assays and contractile activity was measured. Without Bax inhibitor, 66% of the D-iPS cell-derived myotubes were TUNEL-positive. The apoptosis of myotubes was significantly suppressed to 42% and 51% by adding 0.1 and 1.0 µM Bax inhibitor, respectively (Figure 5A,B), although 10 µM Bax inhibitor did not prevent myotube apoptosis. Thus, Bax inhibitor was effective for preventing apoptosis at low concentrations, and high concentration of Bax inhibitor may cause toxic effect for myotubes. In addition, the myotube displacement was 1.8 µm without Bax inhibitor, but was significantly increased to 2.7 and 2.8 µm by adding 0.1 and 1.0 µM Bax inhibitor, respectively (Figure 6A). Furthermore, the efficiency of contracting myotubes was also increased 33% and 37% by adding 0.1 and 1.0 µM Bax inhibitor compared with no Bax inhibitor (Figure 6B). These results indicate that apoptosis of myotubes derived from D-iPS cells in EPS culture was suppressed and that the contractile activity of myotubes was increased by inhibition of the Bax pathway.

## 4. Discussion

Here, we showed that EPS culture is effective in improving the contractile activity of human iPS cell-derived myotubes. Furthermore, we demonstrated that contractile activity can be restored by *DMD* gene repair in iPS cells derived from a DMD patient using CRISPR-Cas9 technology.

Kennedy et al. reported that retinoic acid induced *Pax3* expression in mouse ES cells and P19 cells and improved the efficiency of differentiation into muscle progenitor cells [20]. Pawlowski et al. reported that human iPS cells can differentiate into myotubes by inducing *MYOD1* expression and by the addition of retinoic acid [21]. In this study, retinoic acid effectively induced muscle differentiation of iPS cells derived from a healthy donor, a DMD patient, and *DMD* gene-repaired cells (Figure 1). In contrast, muscle hypertrophy was not induced with retinoic acid. *Pax3* upregulated by retinoic acid enhances *Pax7* expression and induces mesoderm or muscle progenitor cells derived from stem cells, but is not directly involved in gene induction for promoting muscle hypertrophy [20].

Previous studies have reported that EPS culture improves muscle contractile function of C2C12 cell-derived myotubes and primary muscle cells [17,22]. However, EPS culture has not been applied to human iPS cell-derived myotubes. In this study, we found that EPS culture improved the contractile activity of human iPS cell-derived myotubes. Muscle contractile activity is thought to be enhanced in EPS culture by mechanical stimulation and expression of specific genes. Muscle contraction is known to trigger the expression of some myokines, such as decorin [23]. Decorin, which is secreted by muscle, binds to myostatin to promote muscle hypertrophy [24]. Furthermore, expression of myosin heavy chain, which is associated with muscle contraction, was enhanced by EPS culture [17]. Kawahara et al. showed that EPS culture promoted the differentiation of myoblasts into myotubes, but the promotion of muscle differentiation was independent of muscle contractile activity [25]. They also reported that expression of genes involved in muscle maturation, such as myogenin, was maintained in EPS culture [25]. In this study, it is assumed that EPS culture facilitates maturation of non-contracting myotubes, thereby improving the efficiency of contracting myotubes.

In D-iPS cell-derived myotubes, contractile activity was improved by low voltage EPS culture, but apoptotic activity was also increased (Figure 2). This indicates that myotubes from DMD patients are more likely to be damaged under high load conditions, but that mild EPS can improve contractile activity in myotubes from DMD patients. This observation is consistent with other reports of increased oxidative stress in post-exercise muscle tissue in mdx mice compared with wild-type mice [26]. Previous reports suggest that DMD pathology is caused by repeated degradation and regeneration of myofibers and myotubes differentiated from muscle progenitor cells, with a faster rate of apoptotic degradation than of muscle differentiation [6]. Therefore, EPS culture of D-iPS cell-derived myotubes induced severe apoptosis (Figure 3) and did not improve contractile activity under high-voltage EPS culture (Figure 2), reproducing the situation in DMD patients. In contrast gene repair in R-iPS cell-derived myotubes restored dystrophin expression. Thus, the contractile behaviour of R-iPS cell-derived myotubes was similar to that of N-iPS cell-derived myotubes. In addition to apoptosis, necrosis also simultaneously occurs in DMD pathology [27]. Therefore, future studies investigating whether necrosis and apoptosis co-occur in EPS cultures are needed to assess whether the experimental system reported here truly mimics the pathology of DMD.

We hypothesised that apoptosis of D-iPS cell-derived myotubes in EPS culture was caused by excessive Ca^2+^ influx. In DMD, dystrophin deficiency causes Ca^2+^ channels to malfunction causing Ca^2+^ influx [28]. Shoji et al. reported that Ca^2+^ flowed into myotubes derived from D-iPS cells during EPS [9]. This increases the intracellular Ca^2+^ concentration causing mitochondrial damage and apoptosis due to oxidative stress [29]. Indeed, addition of EGTA, a Ca^2+^ chelator, suppressed apoptosis (Figure 4). These results indicate that Ca^2+^ is involved in apoptosis in EPS culture. Thus, regulating Ca^2+^ influx may reduce the symptoms of DMD.

In the apoptotic pathway involving Ca^2+^, Ca ions accumulate in mitochondria and open the mitochondrial permeability transition pores [30,31]. This causes cytochrome C release resulting in apoptosis [30,31]. Bax promotes Ca^2+^ uptake by mitochondria [30,31]. In addition, Bax expression is increased in DMD patient blood samples [32]. In this study, inhibiting the Bax pathway suppressed apoptosis and improved muscle contractile activity (Figure 5 and Figure 6). These results indicate that D-iPS cell-derived myotubes in EPS culture mimic the pathology of DMD patients.

Currently, there is no established way to treat DMD, although exon skipping has recently been reported for DMD treatment [33,34]. In this method, defective DMD exons are skipped producing functional but partially deleted dystrophin, which suppresses apoptosis [33,34]. In contrast, gene correction using genome editing technology has the benefit of restoring expression of the intact dystrophin protein. EPS culture not only improves the contractile activity of human iPS cell-derived myotubes, but also helps to reproduce muscle functionality. The method presented in this study is effective for evaluating the results of gene therapy in terms of the muscular function of cells and provides a useful tool for the development of future DMD therapeutic methods.

In conclusion, we showed that the quality of human iPS cell-derived myotubes can be evaluated from their contractile activity by measuring contractile response to electrical pulse stimulation. As a model, DMD patient-derived iPS cells and iPS cells repaired using CRISPR-Cas9 technology were used for evaluation. The maturity of iPS cell-derived myotubes was improved by adding retinoic acid during differentiation culture, making it possible to measure the contractile activity of myotubes. Using this method, we found that Bax inhibitor alleviated cell damage caused by abnormal Ca^2+^ influx during electrical stimulation in DMD patient-derived myotubes. This method measures contractile activity, which is a direct indicator of skeletal muscle function, and assesses myotube function simply and easily, compared with conventional gene expression-based or physiological methods. This contractile activity-based system can be widely used for myotube evaluation.

## Data Availability

The raw data presented in this study are available from the corresponding author upon request.

## Supplementary Materials

The following are available online at https://www.mdpi.com/article/10.3390/cells10102556/s1, Figure S1: Effect of RA on width of human iPS cell-derived myotubes. Myotubes were differentiated from N-iPS (A), D-iPS (B), and R-iPS (C) cells, Figure S2: Western blot analysis for myogenic markers of human iPS cell-derived myotubes. Human iPS cells (N-iPS, D-iPS and R-iPS) were differentiated into myotubes as described in Section 2.2 with or without 1.0 μM RA. On day 14 of differentiation culture, myotubes were detached from the cultured wells using Accutase in accordance with a standard protocol. After harvesting the cells by centrifugation, cells were washed with PBS twice, suspended in PBS containing 1 mM PMSF (P7626; Sigma-Aldrich), and sonicated for disruption. After removing cell debris by centrifugation, cell lysate samples were applied for Wes automated capillary western blotting (Model No. 004-600A-N001; ProteinSimple, San Jose, CA, USA). Primary antibodies were purchased from Santa Cruz Biotechnology (sc-376157 for MyHC), Abcam (ab7785 for tropomyosin), and Cell Signaling technology (2118 for GAPDH). Secondary antibodies were provided from ProteinSimple (anti-mouse [042–205] and anti-rabbit [042–206]).
