# Supplementary material for: Contractile Activity of Myotubes Derived from Human Induced Pluripotent Stem Cells: A Model of Duchenne Muscular Dystrophy
Source: Cells. 2021 Sep 27;10(10):2556. doi: 10.3390/cells10102556 (PMC8534131; doi:10.3390/cells10102556)
Supplement: Supplementary file 1 [file cells-10-02556-s001.zip › cells-1375689-supplementary.pdf]

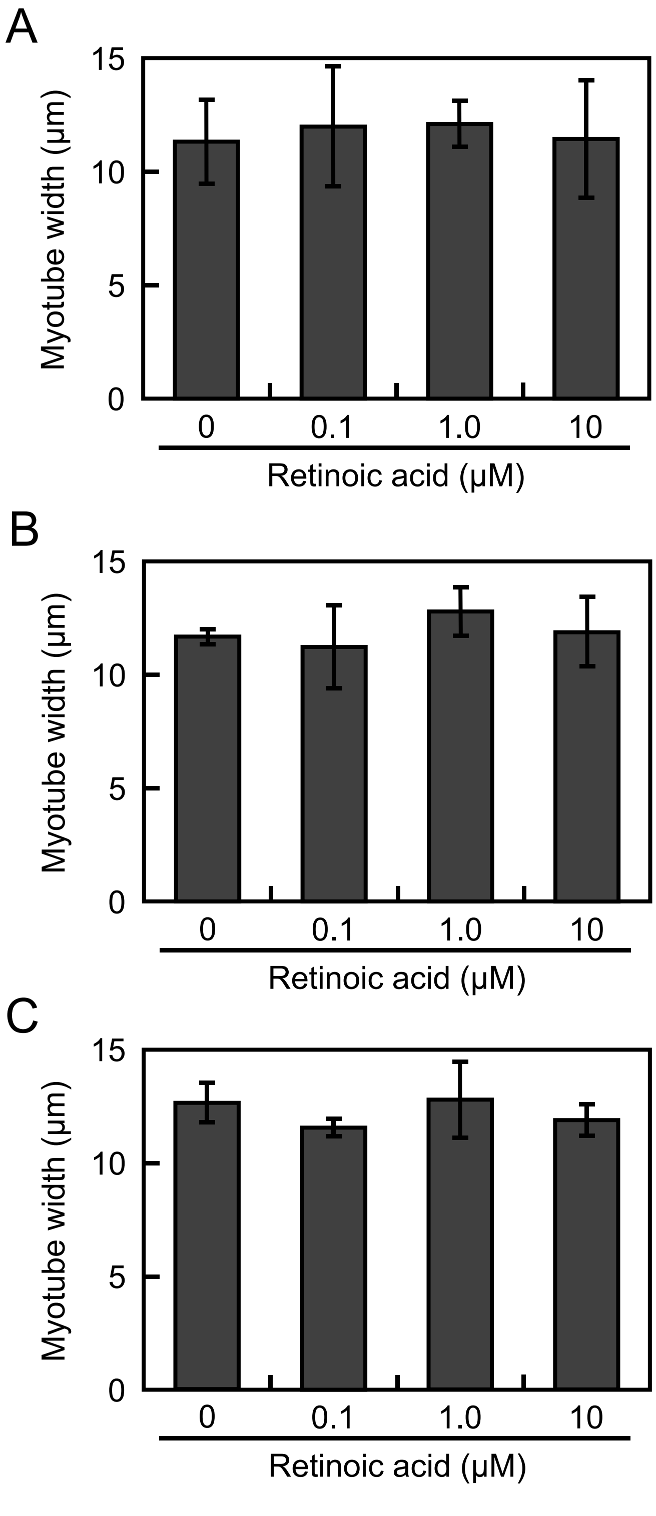


**Supplementary Figure S1.** Effect of RA on width of human iPS cell-derived myotubes. Myotubes were differentiated from N-iPS (A), D-iPS (B), and R-iPS (C) cells.

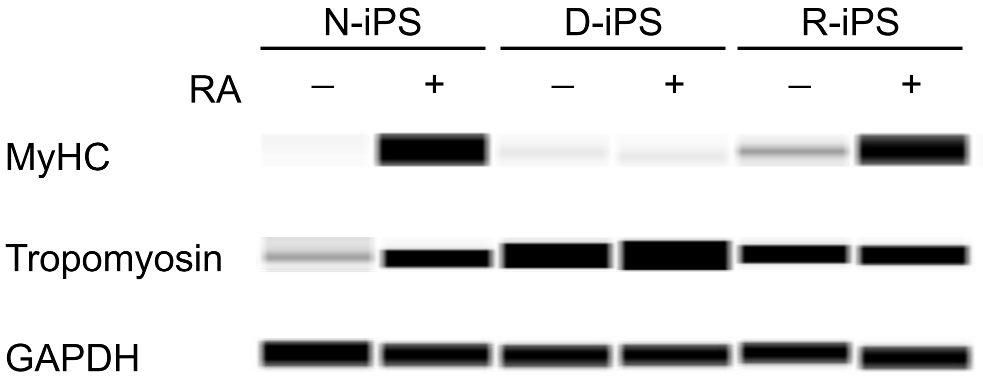


**Supplementary Figure S2.** Western blot analysis for myogenic markers of human iPS cell-derived myotubes. Human iPS cells (N-iPS, D-iPS and R-iPS) were differentiated into myotubes as described in *Myotube differentiation* with or without 1.0 μM RA. On day 14 of differentiation culture, myotubes were detached from the cultured wells using Accutase in accordance with a standard protocol. After harvesting the cells by centrifugation, cells were washed with PBS twice, suspended in PBS containing 1 mM PMSF (P7626; Sigma-Aldrich), and sonicated for disruption. After removing cell debris by centrifugation, cell lysate samples were applied for Wes automated capillary western blotting (Model No. 004-600A-N001; ProteinSimple, San Jose, CA, USA). Primary antibodies were purchased from Santa Cruz Biotechnology (sc-376157 for MyHC), Abcam (ab7785 for tropomyosin), and Cell Signaling technology (2118 for GAPDH). Secondary antibodies were provided from ProteinSimple (anti-mouse [042-205] and anti-rabbit [042-206]).
